# The Prognostic Impact of Comorbidities in Patients with De-Novo Diffuse Large B-Cell Lymphoma Treated with R-CHOP Immunochemotherapy in Curative Intent

**DOI:** 10.3390/jcm9041005

**Published:** 2020-04-02

**Authors:** Florian Kocher, Michael Mian, Andreas Seeber, Michael Fiegl, Reinhard Stauder

**Affiliations:** 1 Department of Internal Medicine V (Hematology and Oncology), Innsbruck Medical University, 6020 Innsbruck, Austria; florian.kocher@i-med.ac.at (F.K.); andreas.seeber@tirol-kliniken.at (A.S.); m.fiegl@pk-hochrum.com (M.F.); 2 Department of Hematology & CBMT, Central Hospital of Bolzano (SABES-ASDAA), 39100 Bolzano-Bozen, Italy; m.mian@med-sci.eu; 3 Riga Stradiņš University, 1007 Riga, Latvia

**Keywords:** comorbidities, HCT-CI, CCI, DLBCL, prognosis, R-CHOP

## Abstract

Background: Patient-related factors, namely comorbidities, impact the clinical outcome of patients with diffuse large B-cell lymphoma (DLBCL). Methods: The prevalence and prognostic impact of comorbidities were examined using the validated scores Charlson Comorbidity Index (CCI) and Hematopoietic Cell Transplantation-specific Comorbidity Index (HCT-CI) in 181 patients with DLBCL at initial diagnosis before treatment with rituximab, cyclophosphamide, vincristine, doxorubicin and prednisone (R-CHOP). Results: Pronounced comorbidities as defined by CCI and HCT-CI scoring of ≥2 were detected in 9.9% and 28.2% of patients, respectively, and occurred more frequently at advanced age (*p* < 0.001). Higher CCI scoring was associated with lower complete response rate (*p* = 0.020). Both advanced CCI and HCT-CI were significantly associated with shortened overall survival (3-year OS: CCI ≥2 vs. 0–1, 38.9% vs. 81.3%, *p* < 0.001; HCT-CI ≥2 vs. 0–1, 56.9% vs. 84.9%, *p* < 0.001). Both comorbidity scores remained independent risk factors in the multivariate analysis (HCT-CI ≥2 HR: 2.6, *p* = 0.004; CCI ≥2 HR: 3.6, *p* = 0.001). Conclusion: This study demonstrates the prognostic relevance of comorbidities classified by CCI and HCT-CI in patients with DLBCL undergoing curative treatment with R-CHOP. A structured evaluation of comorbidities might refine prognostication alongside currently used prognostic parameters, namely age, and should be evaluated in prospective trials.

## 1. Introduction

Diffuse large B cell lymphoma (DLBCL) accounts for approximately 25–30% of all types of non-Hodgkin lymphomas (NHL), thus representing the most common histological subtype [1]. The clinical course of the disease is heterogeneous and is affected by various parameters including comorbidities, malnutrition, impaired functional capacities and reduced resilience and organ reserve [2,3,4]. The International Prognostic Index (IPI) was devised to predict the survival of patients with DLBCL. As the IPI classification was developed in the pre-rituximab era, when predominantly patients below the age of 60 were eligible for intensified treatment regimens, the cutoff for age as a risk factor was set at 60 years [5]. However, with the introduction of rituximab, the treatment of NHL has changed substantially and outcome has improved markedly. Several large trials have shown improved survival in DLBCL patients >60 years using the combination of rituximab, cyclophosphamide, vincristine, doxorubicin and prednisone (R-CHOP) [6,7,8,9,10]. Moreover, the broad availability of granulocyte colony-stimulating factor (G-CSF) has facilitated comparable dose intensities in both older and younger patients [11]. Consequently, guidelines recommend this immune-chemotherapy, even in selected subgroups of patients at advanced age [4,12]. As a consequence of new treatment options and to enhance the prognostic impact, modifications of the IPI have been established including the R-IPI and the NCCN-IPI [13,14]. Nevertheless, the original IPI is still widely used to evaluate prognosis in DLBCL.

The prognostic parameters of the IPI consider the biology and the stage of the malignant disorder, as well as the age and the performance status of patients. Recently, the relevance of patient-related factors, namely comorbidities, were recognized as important parameters for prognostication and decision-making. Therefore, the integration of comorbidity scores into clinical practice has been suggested [12]. Comorbidities represent competing adverse risk factors and affect treatment decision-making in patients since they can preclude the use of certain drugs or require a dose reduction that may lead to a dismal outcome [15]. Several validated scores for assessing comorbidities have been reported in the literature so far [16]. The Charlson Comorbidity Index (CCI) was originally developed to elaborate the prognostic significance of comorbidities irrespectively of the underlying disease [17]. This score has also been used to estimate the comorbidity burden in cancer patients [18], including DLBCL [19,20,21,22]. The Hematopoietic Cell Transplantation-specific Comorbidity Index (HCT-CI) was developed to evaluate the feasibility and the risk of patients undergoing intensive therapy and hematopoietic stem cell transplantation (HSCT) [23,24,25,26]. However, the clinical relevance of the HCT-CI in patients not undergoing HSCT is still a matter of debate and this score has so far not been evaluated in DLBCL patients treated with R-CHOP.

The goal of this study was to analyze the prognostic impact of the comorbidity burden as defined by the CCI and the HCT-CI in real-life in a homogeneous cohort of consecutive DLBCL patients treated at a single institution.

## 2. Methods

### 2.1. Patients and Methods

Between October 2000 and November 2012, 181 consecutive patients newly diagnosed with DLBCL and treated in first line in curative intent with full dose R-CHOP at the Medical University of Innsbruck were included in this study. Parameters included were baseline characteristics, treatment-related variables and clinical outcome. While the clinical data and the outcome of this cohort of patients have already been published [27,28], the present analysis focuses on the prevalence and clinical impact of distinct comorbidities at initial diagnosis. The burden and prognostic impact of comorbidities were evaluated with two validated comorbidity scores that were retrospectively assessed using the patients’ charts. A detailed description of these scores including definitions and grading of comorbidities is provided elsewhere [17,23]. An elevated comorbidity score was defined as either CCI ≥ 2 or HCT-CI ≥ 2.

### 2.2. Statistics

The Chi-square test was performed to assess the significance of differences between categorical variables. Response was assessed with CT or PET-CT applying the international response criteria for NHL [29,30]. Overall survival (OS) was plotted as a curve using the Kaplan–Meier method. The Log-rank test was employed to assess the impact of categorical variables on survival. Multivariate analyses were done according to the Cox regression and the binary logistic regression method. A receiver operating characteristics (ROC) analysis was employed to elaborate the discriminative power of both comorbidity scores. Distributions were compared with the Wilcoxon–Mann–Whitney test. A *p*-value of < 0.05 was considered statistically significant. All statistical analyses were performed with the Statistical Package for the Social Sciences (SPSS) software, version 20.0 (IBM, Armonk, NY, USA). The study was approved by the Ethics Committee of the Medical University of Innsbruck.

## 3. Results

### 3.1. Clinical Characteristics

Altogether, 181 consecutive patients with newly diagnosed DLBCL were included in this analysis. Median age at diagnosis was 60 (SD 16.4) years. Median time between diagnosis and start of treatment with R-CHOP was 16 days (interquartile range 9–28). Of the 181 included patients 85 (47.0%) were female. An IPI ≥ 3 was recorded in 63 (42.6%) of 148 evaluable patients. In total, 96.1% of patients received at least four cycles of R-CHOP. Complete response (CR) on first-line R-CHOP was achieved in 135 (74.6%) patients. A relapse was observed in 53 (30.5%) of 174 patients (Table 1).

### 3.2. Charlson Comorbidity Index

Based on CCI criteria 76.8% of the patients (*n* = 139) did not reveal any comorbidities. 13.3% had one index point and 9.9% had an index ≥ 2. The most prevalent comorbid conditions were chronic obstructive pulmonary disease, diabetes without complications and a secondary non-metastatic tumor (Table 2).

### 3.3. Hematopoietic Cell Transplantation-Specific Comorbidity Index

According to the HCT-CI, 64.1% (*n* = 116) of the patients presented without comorbidities, whereas in 7.7% (*n* = 14) one index point was observed. The remaining 28.2% (*n* = 51) of the patients had at least two HCT-CI points. The most prevalent comorbidities were a history of a previous solid tumor and cardiovascular comorbidities (Table 3).

### 3.4. Association between Comorbidities and Clinical Characteristics

**Figure 1:**
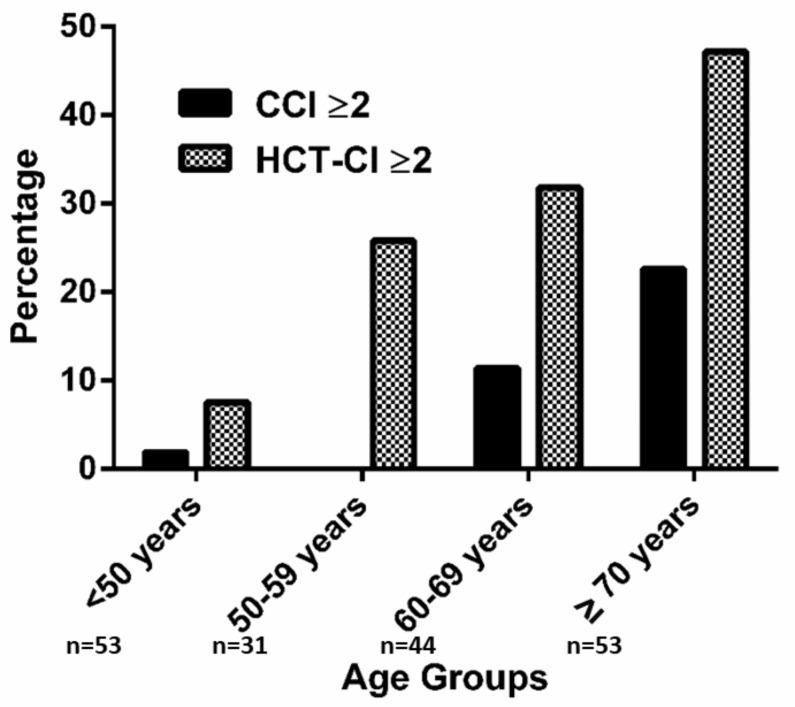
Prevalence of HCT-CI and CCI ≥2 according to age groups. Prevalence rates according to age groups: <50 years: CCI ≥ 2, 1.9% HCT-CI ≥ 2, 7.5%; 50–59 years: CCI ≥ 2, - HCT-CI ≥ 2, 25.8%; 60–69 years: CCI ≥ 2, 11.4% HCT-CI ≥ 2, 31.8%; ≥70years: CCI ≥ 2, 22.6 HCT-CI ≥ 2, 47.2%.

Advanced age was significantly associated with an increased burden of comorbidities: in patients <50 years a CCI ≥ 2 and a HCT-CI ≥ 2 were recorded in 1.9% and 7.5% of patients, whereas in individuals ≥70 years the prevalence was 22.6% and 47.2%, respectively (*p* < 0.001) (Figure 1). In a binary logistic regression model including the parameters of the IPI, only age was significantly associated with a CCI ≥ 2 (*p* = 0.006) and a HCT-CI ≥ 2 (*p* = 0.001).

**Figure 2:**
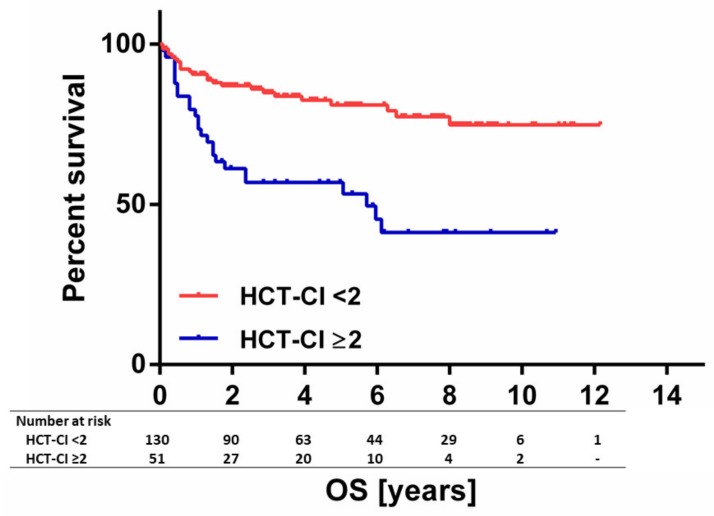
Overall survival according to HCT-CI. 3-year and 5-year OS was 84.9% and 81.0% in patients with an HCT-CI of 0–1. 3-year and 5-year OS was 56.8% and 56.8% in patients with an HCT-CI of ≥2.

The number of administered R-CHOP cycles was comparable between groups with low vs. high comorbidities: (≥4 treatment cycles; HCT-CI: 96.9% vs. 94.0%, *p* = 0.403; CCI: 96.6% vs. 88.9%, *p* = 0.150). Likewise, time between diagnosis and treatment start was comparable between the two comorbidity groups (HCT-CI: *p*= 0.474; CCI: *p* = 0.686). Duration of R-CHOP treatment, which might be considered a surrogate for dose-density, was similar in patients with CCI < 2 and CCI ≥ 2 (*p* = 0.359). When patients were broken down according to HCT-CI, a trend towards prolonged treatment duration was observed for the HCT-CI ≥ 2 group (*p* = 0.092). The complete remission (CR) rate was 77.3% in patients with CCI < 2, whereas the CR rate was 50.0% in patients with CCI ≥ 2 (*p* = 0.020). The CR rate in the HCT-CI groups was 78.5% and 64.7% in HCT-CI < 2 and HCT-CI ≥ 2 patients, respectively (*p* = 0.061) (Supplementary Table S1). With regard to relapse-free survival, defined as time from diagnosis until disease relapse, no significant differences were observed (CCI < 2 vs. CCI ≥ 2: 10.4 vs. 6.9 years, *p* = 0.642; HCT-CI < 2 vs. HCT-CI ≥ 2: 10.2 vs. 9.9 years, *p* = 0.246). At final analysis median OS had not been reached: 3-year and 5-year OS rates were 76.6% and 74.0%, respectively. All parameters of IPI, the number of administered treatment cycles as well as the response to R-CHOP were significantly associated with OS in univariate analysis (Table 4). Most importantly, high comorbidity burden as assessed by both scores, represented an adverse prognostic factor. Patients with a CCI score of 0–1 had a 3-year OS rate of 81.3%, whereas this percentage was significantly lower in patients with CCI ≥ 2: 38.9% (*p* < 0.001) (Table 4). Likewise, the 3-year OS rate was 84.9% and 56.9% in patients with 0–1 and ≥ 2 in HCT-CI, respectively (*p* < 0.001) (Table 4 and Figure 2). In an exploratory ROC analysis to predict death, both comorbidity scores showed a comparable discriminative power (HCT-CI: AUC 0.654, 95%CI 0.56–0.75, *p* = 0.002; CCI: AUC 0.657, 95%CI 0.56–0.75, *p* < 0.001).

As can be seen in Table 4, extranodal site, performance status ≥2, age ≥60 years and both comorbidity scores were included in two Cox-proportional hazard models (*n* = 136). In both multivariate models, advanced stage at diagnosis (≥III), performance status ≥ 2 and either HCT-CI or CCI ≥ 2 remained independent prognostic factors. Thus, HCT-CI ≥ 2 and CCI ≥ 2 were associated with a 2.6 (95% CI 1.4–5.0) and a 3.6 (95% CI 1.7–7.4) -fold increased risk for death (Table 5, Supplementary Table S2).

## 4. Discussion

To date, the IPI has represented the gold standard for prognostication in DLBCL. As a means of refining prognostic scoring the integration of comorbidities has been suggested [4]. This retrospective study aimed to assess the prevalence and the prognostic impact of comorbidity burden as defined by the validated scores CCI and HCT-CI in consecutive patients with DLBCL undergoing standard dose R-CHOP immuno-chemotherapy in curative intent.

Our cohort of patients was characterized by a relevant comorbidity burden, as highlighted by CCI scoring ≥ 2 in 9.9%. This proportion is within the range of 5.8–26% reported in retrospective series in the literature [19,20,21,31]. In a study focusing on patients with DLBCL at advanced age (≥75 years) a CCI ≥ 2 was detected in 34.9% [22], which is slightly above the prevalence of 26.7% in our subgroup [22]. This difference might be explained by the selection of patients in our cohort, who were considered to be fit to tolerate R-CHOP therapy based on evaluation by the treating physicians.

The HCT-CI was developed to better define and assess pre-existing comorbidities in hematological malignancies [23]. The better sensitivity of the HCT-CI as compared to the CCI is supported by a prevalence of 28.8% vs. 9.9% for scoring ≥ 2 in this study. To date, there has been a lack of knowledge concerning the prevalence of HCT-CI-defined comorbidities in patients with DLBCL. In the initial description of the HCT-CI a scoring ≥ 2 was reported in 55% of patients undergoing allogeneic HSCT [23]. The discrepancy as opposed to a lower prevalence of 28.8% in our study might be explained by different patient cohorts and the more profound diagnostic work-up prior to HSCT. For example, moderate pulmonary disease was detected in 24% of HSCT patients, whereas it was observed in only 2.2% of our cohort. While classification of pulmonary comorbidities in our study is based on grading dyspnea [23], the evaluation of patients before HSCT is generally based on more sensitive pulmonary function tests including evaluation of diffusion capacity and expiratory volume. Moreover, the lower prevalence in our study may be explained by retrospective assessment as compared to prospective analyses. In patients with NHL at advanced age (>70 years) and who received autologous HSCT, a HCT-CI grade of 0 was reported in 73% [32]. The percentage in this subgroup in our cohort was 20.7%. Differences may be explained by selection criteria, as patients who were fit for HSCT were selected. In summary, our findings serve as the first benchmark, aside from HSCT, for the prevalence of HCT-CI-defined comorbidities in DLBCL.

Most importantly, an increased comorbidity burden as defined by both scores was an independent predictor of clinical outcome. The prognostic relevance of CCI in this study is in line with results from previous studies conducted in patients with DLBCL [19,31,33,34]. However, to the best of our knowledge this is the first study reporting the relevance of HCT-CI as a poor prognosticator in the setting of R-CHOP-treated DLBCL patients. Based on an exploratory ROC analysis, both comorbidity scores showed comparable discrimination and provide similar prognostic accuracy with regard to overall survival.

Remarkably, advanced age as dichotomized variable in accordance of the IPI definition, was a significant predictor in univariate analyses, but this association was no longer present when comorbidities were included in multivariate analyses. Additionally, the age cut-off set at 70 years failed to be an independent prognostic factor in another multivariate model. However, it has to be mentioned that age remained a significant independent prognostic factor when a model was performed with age as continuous variable. In general, age is a commonly used prognostic parameter in hematological malignancies [4,12,35]. However, a more thorough classification of patients under the aspect of prognosis was demonstrated by including parameters like physical capacity, nutritional status, and comorbidities in several studies in hematological malignancies [35]. Our findings are in line with these observations and suggest that comorbidities should be integrated in prognostic scoring. In fact, Antic et al. developed a modified NCCN-IPI by including the CCI, resulting in an increase in the prognostic value of 2.1% [36]. These findings highlight the relevance of a structured evaluation of comorbidities as an additional independent prognostic factor in DLBCL. The observations that the HCT-CI provides better accuracy in detecting comorbidities and reveals a comparative discriminative power comparable to that of the CCI may form the basis for suggesting that the HCT-CI should be integrated into combined scores.

Clinical outcome in R-CHOP-treated patients with DLBCL is associated with the administered dose intensity [37,38]. Whereas there was no difference in the number of administered therapy cycles, the response rate was significantly improved when comparing low- and high-comorbidity groups. Due to incomplete data analysis on the extent of dose-reductions as additional factor affecting response was not possible. On the other hand, relapse-free survival was not significantly inferior in patients with high comorbidity. The inferior outcome in patients with high comorbidity might be explained in part by a trend towards prolonged treatment duration, a reduced dose intensity as well as an increase in therapeutic interruptions. These findings suggest that integration of comorbidities might be helpful as a means of identifying patients at high risk for treatment toxicity as well as vulnerable patients, in whom attenuated therapy may be a more reasonable treatment option [4,39,40]. The finding that relapse-free survival was not associated with the burden of comorbidity, whereas it was an independent prognostic factor for OS, might reflect that patients with high comorbidities are at increased risk for other causes of death. The strength of the presented work is that it evaluates comorbidities in a well defined cohort of consecutive DLBCL patients treated homogeneously with R-CHOP in curative intent. Moreover, the unicentric design of this analysis ensures the constant validity of data evaluation. This study thus adds relevant information, as analyses published so far have often focused on the impact of comorbidities in DLBCL patients treated with various treatment regimens [19,21,34,36]. A weakness of this study is the retrospective evaluation of comorbidities, which may result in the underestimation of their prevalence. Moreover, the retrospective nature of the study prohibited data acquisition with regard to dose-reductions which might have been a possible reason for lower CR rates in the high comorbidity groups. Additionally, incomplete data precluded further analyses on therapy associated toxicity or death reasons. Future analyses are needed to extend these findings with a focus on prospective assessment and evaluation of both patient- and physician-reported toxicities.

Improved life expectancy will result in an increasing number of cancer patients at advanced age [12,41]. Thus, the prevalence of patients with comorbidities will rise dramatically over the next decades and there will be an urgent need to develop treatment algorithms in cancer patients with multiple co-existing diseases. Attenuated treatment regimens like R-miniCHOP or ofatumumab-miniCHOP have already shown to provide good efficacy and safety in lymphoma patients above 80 years [42,43]. Additionally, standardized comorbidity evaluations might serve as an easy-to-use and inexpensive tool for elaborating patient prognosis and individualizing treatment decisions [44]. Integration of different domains of geriatric assessment will serve to tailor the treatment algorithms in this large group of patients irrespective of currently established age cutoffs [45].

## 5. Conclusions

In patients with DLBCL undergoing treatment with R-CHOP the burden of comorbidities, evaluated by the CCI and HCT-CI, represents an independent adverse prognosticator. Structured evaluations of comorbidities by validated scores might add important information on the prognosis of DLBCL patients aside of currently used prognostic items and should be evaluated in prospective trials.

## Figures and Tables

**Table 1:** Clinical characteristics.

	Valid Cases *n*	Clinical Characteristics *n* (%)
**Baseline characteristics**		
Total number	181	100
Median age (range), years		60 (18–90)
Women	181	85 (47.0)
Age ≥ 60 years		91 (50.3)
B-symptoms	156	65 (41.7)
Ann Arbor Stage	181	
I		29 (16.0)
II		51 (28.2)
III		40 (22.1)
IV		61 (33.7)
>1 Extranodal site	172	52 (30.2)
WHO Performance Status ≥2	155	37 (23.9)
LDH > UNL	160	91 (56.9)
IPI ≥ 3	148	63 (42.6)
Lymphadenopathy >5cm and/or Maximum spleen diameter ≥20cm	166	77 (46.4)
**Treatment**		
1 cycle R-CHOP	178	2 (1.1)
2 cycles R-CHOP		2 (1.1)
3 cycles R-CHOP		3 (1.7)
4 cycles R-CHOP		6 (3.4)
5 cycles R-CHOP		7 (3.9)
≥6 cycles R-CHOP		158 (88.8)
**Response**	181	
CR		135 (74.6)
PR		16 (8.8)
SD		1 (0.6)
PD		20 (11.0)
Interruption		3 (1.7)
Death		5 (2.8)
Unknown		1 (0.6)
Relapses	174	53 (30.5)

**Table 2:** Comorbidities as defined by the Charlson Comorbidity Index (CCI).

Charlson Comorbidity Index	Scoring Points	Comorbidities According to the CCI *n* (%)
Second solid tumor (non-metastatic)	2	7 (3.9)
Diabetes (without complication)	1	7 (3.9)
Chronic pulmonary disease	1	7 (3.9)
Connective tissue disease	1	6 (3.3)
Congestive heart failure	1	4 (2.2)
Peripheral vascular disease	1	4 (2.2)
Cerebrovascular disease (except hemiplegia)	1	4 (2.2)
Ulcer disease	1	4 (2.2)
Diabetes with end organ damage	2	3 (1.7)
Myocardial infarction	1	2 (1.1)
Moderate or severe liver disease	3	2 (1.1)
Mild liver disease	1	1 (0.6)
Hemiplegia	2	1 (0.6)
Moderate or severe renal disease	2	-
Dementia	1	-
Leukemia	2	-
Lymphoma	2	-
Acquired immunodeficiency syndrome	6	-
Second solid tumor (metastatic)	6	-
**Charlson Comorbidity Scoring points**		
CCI 0		139 (76.8)
CCI 1		24 (13.3)
CCI 2		13 (7.2)
CCI 3		3 (1.7)
CCI 4		2 (1.1)

**Table 3:** Comorbidities as defined by the Haematopoetic Cell Transplantation-specific Comorbidity Index (HCT-CI).

HCT Comorbidity Index	Scoring Points	Comorbidities According to the HCT-CI *n* (%)
Previous solid tumor	3	17 (9.4)
Cardiovascular comorbidity	1	13 (7.2)
Renal comorbidity	2	12 (6.6)
Diabetes	1	10 (5.5)
Arrhythmia	1	10 (5.5)
Rheumatologic comorbidity	2	6 (3.3)
Heart valve disease	3	5 (2.8)
Psychiatric disturbance	1	5 (2.8)
Cerebrovascular disease	1	4 (2.2)
Pulmonary comorbidity moderate	2	4 (2.2)
Peptic ulcer	2	4 (2.2)
Obesity	1	3 (1.7)
Severe liver disease	3	2 (1.1)
Pulmonary comorbidity severe	3	1 (0.6)
Mild liver disease	1	1 (0.6)
Inflammatory bowel disease	1	-
Infection	1	-
**HCT-CI Scoring points ** **≥ 2**		
HCT-CI 0		116 (64.1)
HCT-CI 1		14 (7.7)
HCT-CI 2		23 (12.7)
HCT-CI 3		16 (8.8)
HCT-CI 4		6 (3.3)
HCT-CI 5		1 (0.6)
HCT-CI 6		-
HCT-CI 7		4 (2.2)
HCT-CI 8		1 (0.6)

**Table 4:** Prognostic factors for overall survival in univariate analyses.

		Overall Survival		
	Valid Cases *n*	3-Year OS (%)	5-Year OS (%)	*p* Value
Whole cohort	181	76.6	74.0	-
Baseline characteristics				
Gender				
Male	96	81.3	78.1	0.112
Female	85	72.8	69.0	
Age *				
Age <60 years	90	84.4	82.3	<0.001
Age ≥60 years	91	69.0	65.8	
Ann Arbor Stage*				
I-II	80	90.9	90.9	<0.001
III-IV	101	64.7	59.6	
LDH *				
Normal	69	90.9	88.9	<0.001
>UNL	91	67.6	64.4	
WHO Performance Status *				
0–1	118	82.6	80.0	<0.001
≥2	37	57.8	57.8	
Extranodal sites *				
0–1	120	82.2	79.8	0.040
≥2	52	63.3	59.6	
IPI				
<3	85	91.2	89.4	<0.001
≥3	63	56.4	53.7	
Treatment				
≥ 4 cycles R-CHOP	7	33.3	33.3	<0.001
<4 cycles R-CHOP	171	78.1	75.4	
Response				
CR or PR	151	88.1	85.1	<0.001
Other	30	8.2	8.2	
CCI *				
0–1	163	81.3	78.3	<0.001
≥2	18	38.9	38.9	
HCT-CI *				
0–1	130	84.9	81.0	<0.001
≥2	51	56.8	56.8	

* parameters included in multivariate analyses.

**Table 5:** Prognostic factors for overall survival in multivariate analyses using the HCT-CI.

	HR (95% CI)	*p* Value
Ann Arbor Stage ≥ III	4.3 (1.8–10.6)	0.001
Performance Status ≥ 2	2.2 (1.1–4.2)	0.023
HCT-CI ≥ 2	2.6 (1.4–5.0)	0.004
elevated LDH	2.1 (0.8–5.2)	0.129
≥2 extranodal sites	1.1 (0.6–2.3)	0.722
age ≥ 60years	2.0 (0.9–4.4)	0.73

## Supplementary Materials

The following are available online at https://www.mdpi.com/2077-0383/9/4/1005/s1, Table S1: Clinical response compared to comorbidity scoring, Table S2: Prognostic factors for overall survival in multivariate analyses using the Charlson CI.
