# Supplementary material for: The Prognostic Impact of Comorbidities in Patients with De-Novo Diffuse Large B-Cell Lymphoma Treated with R-CHOP Immunochemotherapy in Curative Intent
Source: J Clin Med. 2020 Apr 2;9(4):1005. doi: 10.3390/jcm9041005 (PMC7230391; doi:10.3390/jcm9041005)
Supplement: Supplementary file 1 [file jcm-09-01005-s001.pdf]

Supplementary Materials:

Table S1. Clinical response compared to comorbidity scoring

	**Charlson Comorbidity Index**			**Haematopoietic Cell Transplantation-specific Comorbidity Index**		
	**CCI <2 n (%)**	**CCI ≥2 n (%)**	**P value**	**HCT-CI <2 n (%)**	**HCT-CI ≥2 n (%)**	**p value**
**Response**			0.004			0.123
CR	126 (77.3)	9 (50.0)		102 (78.5)	33 (64.7)	
PR	13 (8.0)	3 (16.7)		12 (9.2)	4 (7.8)	
SD	1 (0.6)	-		1 (0.8)	-	
PD	18 (11.0)	2 (11.1)		12 (9.2)	8 (15.7)	
Interruption	2 (1.2)	1 (5.6)		1 (0.8)	2 (3.9)	
Death	2 (1.2)	3 (16.7)		2 (1.5)	3 (5.9)	
Unknown	1 (0.6)	-		-	1 (2.0)	
**Duration of R-CHOP therapy (days)**	122	133	0.359	119	133	0.092

Table S2. Prognostic factors for overall survival in multivariate analyses using the Charlson CI

	**HR (95% CI)**	**p value**
**Stage ≥III**	4.5 (1.9-11.1)	0.001
**Performance Status ≥2**	2.3 (1.2-4.4)	0.014
**CCI ≥2**	3.6 (1.7-7.4)	0.001

*Parameters included were: LDH, >1 extranodal site, stage, ECOG PS, age ≥60years, CCI. Analyses were performed in 136 patients.
